# Ocular parameters associated with visual performance of enhanced monofocal intraocular lens

**DOI:** 10.1186/s12886-024-03316-w

**Published:** 2024-02-19

**Authors:** Da Ran Kim, Young Chae Yoon, Woong-Joo Whang, Ho Sik Hwang, Kyung-Sun Na

**Affiliations:** Department of Ophthalmology, College of Medicine, The Catholic University of Korea, Seoul, Republic of Korea

**Keywords:** Enhanced monofocal intraocular Lens, Spherical aberration, Angle alpha, Anterior chamber depth, Quality of vision

## Abstract

**Background:**

An enhanced monofocal intraocular lenses (IOLs) (Tecnis Eyhance ICB00 and Tecnis Eyhance Toric DIU) has been developed to enhance intermediate vision while avoiding the disadvantages of multifocal IOLs. Although many studies have demonstrated the improvement of intermediate visual acuity with enhanced monofocal IOLs, it is not known specifically for which patients these IOLs should be recommended or avoided. In this study, we aim to find out which ocular parameters affect vision performance and photic phenomenon of ICB00 or DIU at different distances.

**Methods:**

Patients who underwent cataract surgery with ICB00 or DIU, performed by a single surgeon, were included. Before surgery, the patients’ age, gender, axial length, anterior chamber depth, spherical aberration Z (4,0), vertical coma, horizontal coma, angle kappa (κ), angle alpha (α), and other ocular parameters were investigated. One month after surgery, uncorrected near visual acuity (UNVA at 40 cm), uncorrected intermediate visual acuity (UIVA at 66 cm), uncorrected distance logMAR visual acuity (UDVA), IOL decentration, and quality of vision (QoV) questionnaires were conducted.

**Results:**

A total of 43 patients (58 eyes) were included. The results of the univariate linear regression analyses showed a negative correlation between spherical aberration and logMAR UNVA and UIVA (*p* = 0.003, β=-0.51 and *p* = 0.018, β=-0.23, respectively) and a positive correlation between angle α and logMAR UIVA (*p* = 0.036, β = 0.19). Deeper anterior chamber depth (ACD) was associated with poorer total QoV (*p* = 0.018, β = 14.43), particularly in glare, halo, blur, and fluctuation perception. A higher degree of IOL decentration tended to decrease UNVA and UIVA (Pearson correlation coefficient, *r* = 0.336 and *r* = 0.221, respectively); however, no significant effect was observed on UDVA (Pearson correlation coefficient, *r* = 0.042).

**Conclusions:**

In enhanced monofocal IOLs, a higher level of spherical aberration is associated with better performance in UNVA and UIVA, whereas a larger angle α has a negative impact. A deeper ACD negatively affects the QoV.

## Background

A general monofocal intraocular lens (IOL) requires viewing spectacles for near objects. Recently, as the demand for near and intermediate vision has increased due to the rise in use of tablets and smartphones, multifocal IOLs have been developed to improve near, intermediate, and distance visual performances without spectacles. However, the disadvantage of multifocal IOLs is that they have poor contrast sensitivity and can increase visual impairments, such as glare or halo, especially when driving at night [1–3]. A study has shown that visual acuity decreases as the multifocal IOL (Tecnis® ZMB00) decenters inferiorly in patients with myopia [4].

An enhanced monofocal IOL (Tecnis® Eyhance ICB00 and Tecnis® Eyhance Toric DIU) has been developed to enhance intermediate vision while avoiding the disadvantages of multifocal IOLs, such as reduced contrast sensitivity or photic phenomena. This IOL is designed to enhance intermediate distance vision by utilizing high-order aspherical refraction technology to bring about a power change of approximately 0.5 D in the central 2 mm portion of the IOL [5]. Owing to these characteristics, the use of ICB00 and DIU is increasing in South Korea, and research is actively being conducted.

Many studies have found that intermediate visual acuity is enhanced when ICB00 is inserted compared to a general monofocal IOL [6–9]. Meanwhile, photic phenomena such as glare or halo and contrast sensitivity were found to be comparable to monofocal and more improved than the extended depth of focus (EDOF) [10–12]. However, to our knowledge, no studies exist on the preoperative ocular parameters that affect the quality of near, intermediate, and distance visual acuities of the ICB00 or DIU. In particular, considering the characteristics of the lens that improves middle-range vision through a 0.5 D change in power in the central 2 mm area, we tried to determine whether there is an objective or subjective effect on distance, intermediate, and near visual acuity when the intraocular lens is displaced in patients with ICB00 or DIU implantation.

## Methods

### Study population

This retrospective study included patients who underwent cataract surgery with either ICB00 or DIU IOLs implanted between March 1, 2022, and April 30, 2023, at Yeouido St. Mary’s Eye Hospital. Eyes with irregular astigmatism, retinal disease, history of previous ocular surgery, eventful surgery (e.g., anterior capsule tear), or postoperative complications were excluded. This study was approved by the Institutional Review Board (IRB) of Yeouido St. Mary’s Hospital (IRB number: SC23RISI0056), and proceeded in compliance with the declaration of Helsinki. An informed consent was obtained from each participant.

### Preoperative examination

The preoperative ocular parameters were also investigated. The spherical equivalent (SE) was measured using a refractometer (KR-800 A; Topcon, Tokyo, Japan). The anterior chamber depth (ACD) and axial length (AL) were measured using an IOLMaster 700 (Carl Zeiss Meditec AG, Jena, Germany). White-to-white (WTW), spherical aberration (SA) Z (4,0), vertical coma, horizontal coma, angle kappa, and angle alpha were measured using a Pentacam (Oculus Optikgeräte GmbH, Wetzlar, Germany). Angle kappa is the difference angle between the pupillary and visual axes, and angle alpha is the angle between the optical and visual axes. The IOL power was calculated through the SRK/T, Hagis, and KANE formula and selected as a target between 0 and − 0.5 D.

### Surgical technique

All the cataract surgeries were performed by a single operator (N.K.S.). The IOL power was calculated using the SRK/T, Hagis, and KANE formulas. Preoperative astigmatism WTR 1.5 D or more and ATR 0.75 D or more were corrected with DIU. For astigmatism correction, the angle of lens insertion was calculated using the KANE formula. After marking with a toric marker at 0 and 180 degrees in the sitting position before surgery, the steep Keratometry (K) angle was marked once more before the first incision in the lying position. Before surgery, all eyes were instilled with 0.5% proparacaine hydrochloride (Alcaine; Alcon Laboratories Inc., Fort Worth, TX, United States). After making a 2.3 mm temporal corneal incision, continuous curvilinear capsulorhexis was performed. Phacoemulsification was performed using the phaco-chop technique. Posterior capsule polishing was then performed. After folding and loading the IOL as described in the information of use, it was inserted into the posterior capsule. All patients received 0.5% moxifloxacin (Vigamox; Novartis, Seoul, South Korea) and topical 1% prednisolone acetate (Predbell; Jonggeundang, Seoul, South Korea) four times daily and 0.1% bromfenac sodium (Bronuck; Taejoon Pharm, Seoul, Korea) 2 times daily after surgery for a month.

### Postoperative evaluation

**Fig. 1:**
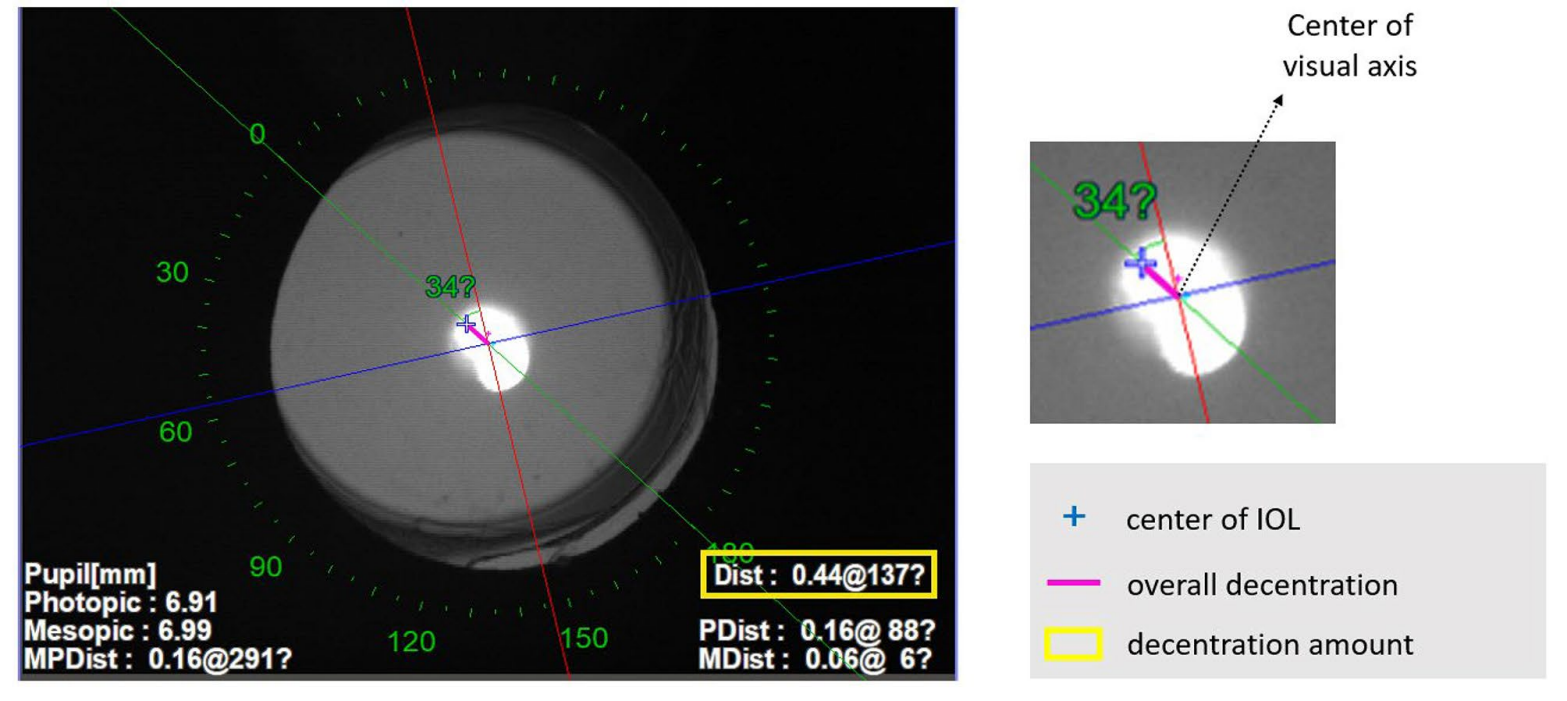
Retrobulbar illumination analysis mode of the OPD-scan III aberrometer. The intersection of the blue and red lines is the visual axis. The blue cross indicates the center of the IOL. The pink line represents the overall decentration connecting the IOL center and the visual axis. The decentration amount is displayed as a yellow box on the lower right

One month after cataract surgery, monocular uncorrected near visual acuity (UNVA) at 40 cm, uncorrected intermediate visual acuity (UIVA) at 66 cm, and uncorrected distance logMAR visual acuity (UDVA) of the IOL implanted eyes were measured. IOL decentration amount was obtained using an OPD scan III aberrometer (Nidek Co., Ltd., Gamagori, Japan) (Fig. 1) [4]. Estimated lens position (ELP) was calculated with ACD, AL, K, IOL power, and postoperative refraction error values. A Quality of vision (QoV) questionnaire was examined based on the criteria proposed by McAlinden et al [13]. 30-item instrument on three scales providing a QoV score in terms of symptom frequency, severity, and bothersome. Regarding how often severe or troublesome photic phenomena such as glare and halo were subjected to a high score on a scale of 0–3. The terminology for each photic phenomenon is explained by the photographs provided in this study.

### Statistical analysis

The correlation between various ocular parameters and visual acuity after surgery was analyzed by performing univariate-adjusted logistic regression analysis using R software (version 4.3.1, R Core Team, Vienna, Austria). The correlation between ocular parameters and the quality of visual acuity was also performed using univariate-adjusted logistic regression analysis with R software in the same way. The relationship between the decentration amount of the IOL and postoperative visual acuity was analyzed by Pearson analysis using SPSS® (Version 26.0, SPSS Inc., Chicago, IL, USA). Statistical significance was set at *p* < 0.05. ACD and angle alpha were divided by compartment according to ascending order to check the t-test, and the section with *P* < 0.05 was checked to find the cut-off, and SPSS was also used.

## Results

A total of 58 eyes from 44 patients who underwent cataract surgery without complications were included in this study: 37 eyes from 28 patients who underwent ICB00 implantation and 21 eyes from 16 patients who underwent DIU implantation. About 24 females (54.5%) and 20 males (45.4%) were enrolled, and their mean age was 65.6 ± 8.8 years (range 39–80 years). The mean preoperative value of the SE was + 0.05 ± 2.61 D (range − 8.5, + 4.0 D), and the corrected distance visual acuity (CDVA) value was 0.40 ± 0.22 logMAR (range 0.05, 1.0 logMAR). The preoperative mean value of AL was 23.91 ± 1.13 mm (range 21.62, 27.03 mm), the ACD value was 3.56 ± 0.41 mm (range 2.62, 4.28 mm), and the WTW value was 11.51 ± 0.32 mm (range 10.8, 12.1 mm). The mean value of SA was 0.35 ± 0.17 (range − 0.274, 0.741), the vertical coma value was 0.00 ± 0.30 (range − 0.717, 0.828 mm), and the horizontal coma value was 0.02 ± 0.20 (range − 0.68, 0.77). The mean value of angle kappa was 0.28 ± 0.11 mm (range 0.07, 0.50), and the mean value of angle alpha was 0.46 ± 0.32 mm (range 0.12, 1.17) (Table 1).

One month after surgery, the mean value of postoperative SE was − 0.76 ± 0.37 D (range − 1.75, 0.12, median − 0.75). The mean value of postoperative UNVA was 0.30 ± 0.15 log MAR, UIVA was 0.10 ± 0.08, and UDVA was 0.10 ± 0.12. The postoperative average value of the IOL decentration amount was 0.25 ± 0.17 mm (range 0.04, 0.63 mm, median 0.22 mm) (Table 1).

**Table 1:** Patient characteristics

Parameters	Value (Mean ± SD)	Range	median
Sex, Male: Female (%)	20 (45.4): 24 (54.5)		
Age, y	65.6 ± 8.8	39, 80	66
SE, D	+ 0.05 ± 2.61	-8.5, +4.0	0.25
CDVA, logMAR	0.40 ± 0.22	0.05, 1.0	0.4
AL, mm	23.91 ± 1.13	21.62, 27.03	23.94
ACD, mm	3.56 ± 0.41	2.62, 4.28	3.61
WTW, mm	11.51 ± 0.32	10.8, 12.1	11.4
Spherical aberration, microm	0.35 ± 0.17	-0.274, 0.741	0.343
Vertical coma(RMS), microm	0.22 ± 0.20	0.006, 0.828	0.166
Horizontal coma(RMS), microm	0.14 ± 0.13	0.002, 0.773	0.118
Angle kappa, mm	0.28 ± 0.11	0.07, 0.50	0.26
Angle alpha, mm	0.46 ± 0.32	0.12, 1.17	0.35
**Post 1 m**			
SE, D	-0.76 ± 0.37	-1.75, 0.12	-0.75
ELP, mm	5.05 ± 0.62	4.18, 7.89	4.99
UNVA, logMAR	0.30 ± 0.15	0.1, 0.6	0.30
UIVA, logMAR	0.10 ± 0.08	0, 0.3	0.10
UDVA, logMAR	0.10 ± 0.12	0, 0.5	0.10
Decenter amount, mm	0.25 ± 0.17	0.04, 0.63	0.22

*SE* Spherical equivalent, *CDVA* Corrected distant visual acuity, *logMAR* Logarithm of the minimum angle of resolution, *AL* Axial length, *ACD* Anterior chamber depth, *WTW* White-to-white, *RMS* root mean square, *ELP* estimated lens position, *UNVA* Uncorrected near visual acuity, *UIVA* Uncorrected intermediate visual acuity, *UDVA* Uncorrected distant visual acuity

In the correlation between visual acuity and ocular parameters, as the SA Z (4,0) increased, UNVA and UIVA improved significantly (β=-0.517, *P* = 0.003, and β=-0.239, *P* = 0.018, respectively). However, no statistically significant difference was observed between Z (4,0) and UDVA (β = 0.194, *P* = 0.098). At the same time, there was no correlation between SA and postoperative SE (Pearson analysis, *P* = 0.176). In addition, as the angle alpha increased, UIVA significantly deteriorated (β = 0.193, *P* = 0.036). The cut-off value of angle alpha for UIVA was 0.35 mm (*p* = 0.02). Although the correlation was slight, the UNVA of females was statistically better than males (β = 0.150, *P* = 0.005). Additionally, as the residual refraction value at 1 month after surgery became more myopic, UDVA decreased statistically significantly (β=-0.514, *P* = 0.002) (Table 2).

**Table 2:** Correlation between ocular parameters and visual acuity

parameters	Visual acuity					
	Near		Intermediate		Distance	
	β	*P*	β	*P*	β	*P*
Sex	**0.150**	0.005*	0.020	0.520	-0.058	0.099
Age, y	0.002	0.510	0.001	0.461	0.002	0.151
WTW, mm	0.103	0.278	0.096	0.076	-0.061	0.352
AL, mm	0.007	0.785	-0.006	0.684	-0.017	0.296
ACD, mm	0.040	0.576	-0.023	0.556	-0.065	0.129
ELP, mm	-0.130	0.478	-0.032	0.864	-0.136	0.350
Spherical aberration, microm	**-0.517**	0.003*	**-0.239**	0.018*	0.194	0.098
Vertical coma(RMS), microm	0.031	0.864	-0.115	0.523	0.156	0.275
Horizontal coma(RMS), microm	-0.146	0.409	-0.194	0.280	0.151	0.292
Angle kappa, mm	0.928	0.088	0.463	0.160	-0.117	0.222
Angle alpha, mm	0.261	0.145	**0.193**	0.036*	-0.060	0.097
Post SE, D	0.224	0.256	0.187	0.334	**-0.514**	0.002*

*WTW* White-to-white, *AL* Axial length, *ACD* Anterior chamber depth, *ELP* estimated lens position, *RMS* root mean square, *SE* spherical equivalent, *Statistically significant

Analysis of the correlation between the quality of visual acuity and each ocular parameter showed that the longer the AL, the higher the total QoV (β = 5.43, *P* = 0.04), indicating that the greater the myopia, the worse the quality of vision. In particular, the deeper the ACD, the higher the total QoV (β = 14.43, *P* = 0.01), glare (β = 2.86, *P* = 0.03), halo (β = 2.25, *P* = 0.02), blurred vision (β = 3.10, *P* = 0.01), and fluctuation scores (β = 1.74, *P* = 0.03) were statistically significant. In this regard, the cutoff value of the ACD for the total QoV score was 3.4 mm (*P* = 0.004). Moreover, a larger SA was associated with more pronounced distortion (β = 3.23, *P* = 0.03), and a larger ELP was associated with worsened total QoV score (β = 0.623, *P* = 0.01), hazy vision (β = 0.74, *P* = 0.00), and distortion (β = 0.52, *P* = 0.02). Neither vertical nor horizontal comas showed statistical significance in QoV. A direct correlation was observed between younger age and increased double image (β=-0.12, *P* = 0.01), fluctuation (β=-0.09, *P* = 0.003), and focusing difficulty scores (β=-0.13, *P* = 0.006). Lastly, as the postoperative SE value became hyperopic, focusing difficulty increased (β = 0.42, *P* = 0.04) (Table 3).

**Table 3:** Correlation between ocular parameters and quality of visual acuity

parameter	QOV(quality of vision)																					
	QoV score		glare		halo		starburst		Hazy vision		Blurred vision		distortion		Double image		fluctuation		Focusing difficulty		Depth perception	
	β	*P*	β	*P*	β	*P*	β	*P*	β	*P*	β	*P*	β	*P*	β	*P*	β	*P*	β	*P*	β	*P*
Sex	0.34	0.94	-1.61	0.16	-0.84	0.34	-0.61	0.26	-0.09	0.91	-1.36	0.20	0.48	0.32	1.88	0.05	0.39	0.57	1.16	0.24	0.96	0.22
Age, y	-0.48	0.06	0.03	0.56	0.04	0.33	-0.02	0.30	-0.04	0.32	-0.05	0.36	-0.02	0.33	-0.12	0.01*	-0.09	0.003*	-0.13	0.006*	-0.06	0.10
WTW, mm	11.82	0.18	1.92	0.35	-0.05	0.97	0.84	0.28	0.66	0.65	0.77	0.66	-0.44	0.6	2.21	0.17	2.09	0.06	1.27	0.42	2.52	0.07
AL, mm	5.43	0.04*	0.97	0.11	0.68	0.15	0.16	0.57	0.61	0.16	0.79	0.17	0.33	0.2	0.40	0.45	0.58	0.11	0.41	0.44	0.44	0.29
ACD, mm	14.43	0.01*	2.86	0.03*	2.35	0.02*	-0.31	0.64	0.52	0.61	3.10	0.01*	0.42	0.48	1.16	0.35	1.74	0.03*	2.07	0.08	0.49	0.61
ELP, mm	0.623	0.01*	0.06	0.82	0.15	0.58	0.46	0.05	0.74	0.00*	0.46	0.07	0.52	0.02*	0.22	0.42	0.43	0.11	0.47	0.07	0.51	0.05
Spherical aberration, microm	-7.62	0.65	-1.39	0.71	1.15	0.69	2.25	0.20	-1.97	0.46	-3.06	0.38	3.23	0.03*	-3.95	0.22	-3.17	0.14	-0.28	0.93	-0.42	0.86
Vertical coma(RMS), microm	0.74	0.46	-0.61	0.54	-0.08	0.74	0.31	0.14	0.26	0.17	0.29	0.21	0.29	0.14	-0.10	0.66	0.06	0.77	0.25	0.28	0.07	0.75
Horizontal coma(RMS), microm	-0.17	0.86	0.58	0.56	-0.1	0.72	-0.32	0.19	0.08	0.71	0.44	0.11	0.26	0.26	0.03	0.91	0.13	0.63	0.30	0.26	0.21	0.43
Angle kappa, mm	-19.90	0.62	-1.65	0.65	-5.25	0.25	-6.38	0.25	-0.15	0.95	0.97	0.59	-2.25	0.53	-2.55	0.77	1.05	0.90	-4.65	0.53	0.97	0.59
Angle alpha, mm	-5.14	0.85	-0.61	0.83	-1.92	0.49	-2.88	0.49	-0.61	0.83	0.35	0.83	-1.92	0.49	0.70	0.83	2.01	0.62	-0.61	0.83	0.35	0.83
Post SE, D	0.28	0.17	0.05	0.80	0.07	0.73	-0.03	0.87	0.07	0.71	0.17	0.42	-0.10	0.62	0.29	0.15	0.34	0.10	0.42	0.04*	0.29	0.16

*WTW* White-to-white, *AL* Axial length, *ACD* Anterior chamber depth, *ELP* estimated lens position, *RMS* root mean square, *SE* spherical equivalent, *Statistically significant

**Fig. 2:**
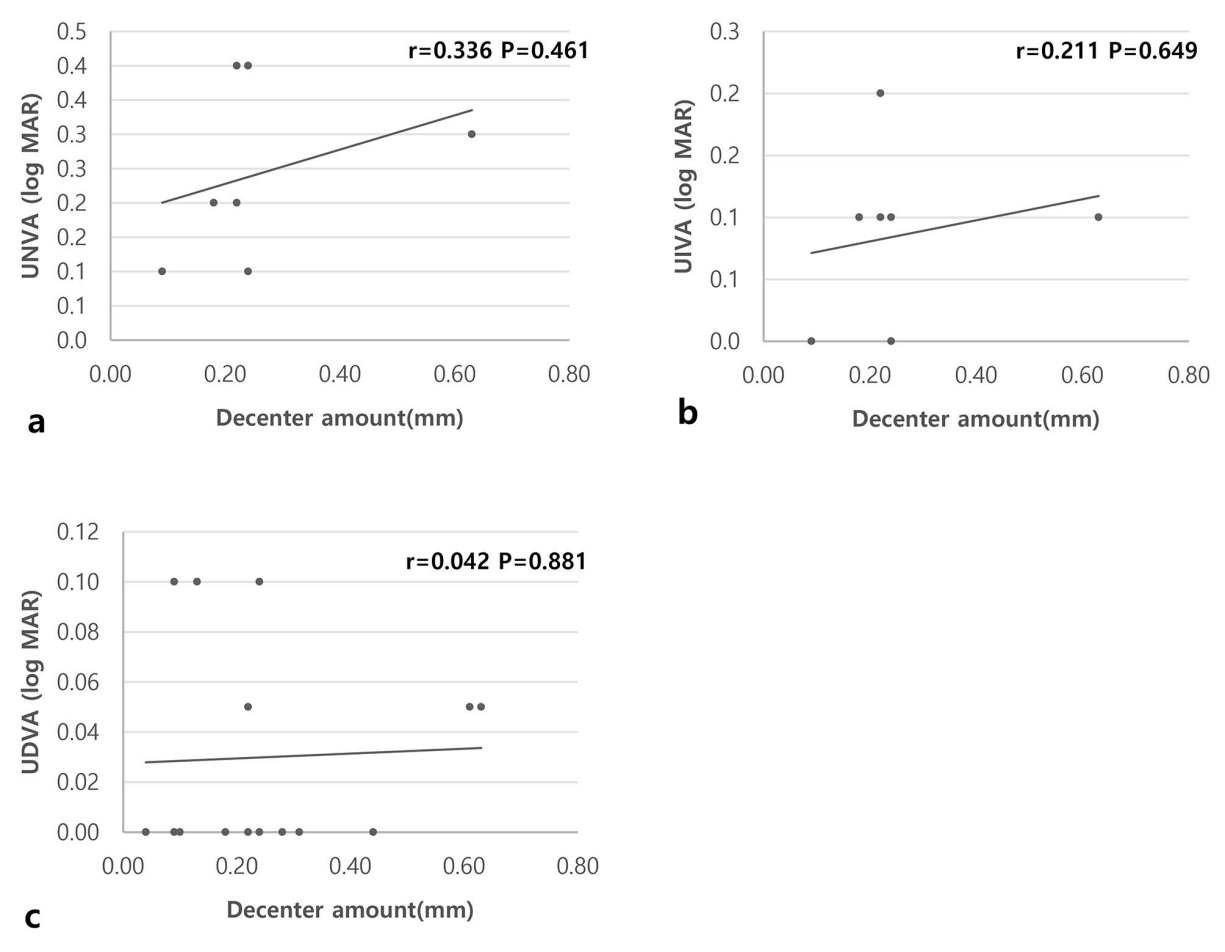
**a** The correlation between the decentration amount of IOL and postoperative UNVA. **b** The correlation between the decentration amount of IOL and postoperative UIVA. **c** The correlation between the decentration amount of IOL and postoperative UDVA

The degree of IOL deviation and postoperative UIVA showed a negative tendency (Pearson analysis, *r*=-0.325, *P* = 0.475) (Fig. 2B), and UNVA also had a negative tendency (Pearson analysis, *r*=-0.452, *P* = 0.308) (Fig. 2A), although the difference was not statistically significant. However, UDVA did not correlate with IOL deviation (Pearson analysis, *r*=-0.003, *P* = 0.992) (Fig. 2C).

## Discussion

In this study, we attempted to determine factors that should be considered before inserting ICB00 or DIU. In this study, SA had a statistically significant effect on the UNVA and UIVA of ICB00, and an increase in SA corresponded to an improvement in UNVA and UIVA (Table 2). Vega et al. [5] and Schmid et al. [14] showed an optical bench study result that the center aspheric design of ICB00 might induce negative SA, affecting myopic shift and depth of field. Several studies have shown that positive and negative changes in SAs increase the depth of field [15, 16]. de Luis Eguileor et al. [17] identified a significant correlation between SA and UIVA, endorsing that with SA of intermediate values, the ICB00 offers better UIVA, which is consistent with the results of this study. Most recently, Abd Elghaffar Shehata et al. [18] found that postoperative ICB00 SA ranging between 0.2 and 0.25 gives the minimum reading add (+ 0.75 D).

Moreover, the postoperative UIVA of ICB00 worsened significantly as the preoperative angle alpha increased (Table 2). Angle alpha is the angle between the optical and visual axes. Although no study exists on the effect of angle alpha in ICB00, recent opinions are available that angle alpha affects dissatisfaction with multifocal IOL [19].

In particular, according to Wang et al. [20], unlike angle kappa, which has a large difference between the preoperative and postoperative values, angle alpha is in the limelight as a preoperative predictor because its preoperative and postoperative values are relatively constant. Lee et al. [21] reported a significant correlation between angle alpha and CDVA in diffractive quadrifocal IOL. For Tecnis EDOF IOLs, Qin et al. [22] found that angle alpha did not affect the visual acuity; however, the value of 0.4 mm or higher in angle alpha affected the visual quality under scotopic conditions and the occurrence of photic phenomena. Given its correlation with ACD and AL, ELP also seems associated with photic phenomena due to its larger size.

Regarding the QoV, deep ACD had a significant effect on total QoV, glare, halo, blurred vision, and fluctuation; the longer the AL, the worse the total QoV (Table 3). The ACD of patients with myopia is deep, and the pupil size is relatively large [23, 24]. Although the preoperative pupil size was not measured in this study, the photic phenomenon was more common in myopic eyes with deep ACD and long AL, likely due to the relatively larger pupil size.

Furthermore, younger age was associated with increased complaints of double images, fluctuations, and focusing difficulty (Table 3). According to de Vries et al. [1], the average age of dissatisfaction with multifocal IOLs was 59.2 ± 12.3 years, which is relatively young. Guillon et al. [25] showed that the pupil size was significantly larger at low luminance and younger ages. Therefore, a younger age results in a heightened susceptibility to photic phenomena, primarily due to the influence of pupil size. Moreover, younger individuals are more prone to situations like night driving or night shifts; thus, increasing their likelihood of experiencing photic phenomena more frequently.

Residual refraction error after surgery can have a significant impact on visual acuity and quality. The mean value of postoperative SE in this study was − 0.76 ± 0.37 D (range − 1.75, 0.12, median − 0.75) (Table 1), slightly myopic, but did not affect UNVA and UIVA. Therefore, it appears that there was no effect of postoperative SE on the results of SA or angle alpha that affected UNVA and UIVA. In a study by Kim et al. [26], it was reported that when mini-monovision of about − 0.75D was provided in binocular ICB00, UDNA was improved while UDVA and UIDA were maintained to a level comparable to binocular emmetropia. However, this is the result when there is binocular summation with a small difference of -0.75D. When considering monocular performance in the range of -1.75D to + 0.12D as in this study, UDVA decreased as it became myopic (Table 2), and focusing difficulty appeared as it became hyperopic (Table 3).

Lastly, as ICB00 decentered, UNVA and UIVA showed moderate tendencies, although this was not statistically significant (Pearson analysis, *r*=-0.452, *P* = 0.308; Pearson analysis, *r*=-0.325, *P* = 0.475, respectively) (Fig. 2A, B). Zhu et al. [4] reported that multifocal IOLs can negatively affect visual acuity when they decenter in the capsular bag. In the case of ICB00, Optical Bench demonstrated that the MTF and Strehl ratio markedly decreased as ICB00 decentered [27]; however, no reports exist in clinical practice. Here, we presented, for the first time in clinical practice to our knowledge, a discernible trend indicating a correlation between ICB00 decentration and poor UIVA and UNVA, despite the absence of statistically significant results. The optical bench study published by Schmid et al. [27] investigated the decentration of 1 mm. In actual clinical practice, the maximum IOL decentration amount was 0.63 mm, the average value was 0.25 ± 0.17 mm, and no significant deviation was observed (Table 1). In a way, the ICB00 seems more robust to decentration than other multifocal IOLs.

Our limitations were the short observation period of 1 month and the small number of patients. In particular, the number of IOL decentrations measured was small; therefore, a limitation was present in confirming the statistical significance. The maximum AL in our study was 27.03 mm, and we rarely included extremely high myopia, resulting in a high IOL decenter amount [4]. Follow-up studies, including more diverse ALs, are needed to confirm a definite association with the IOL decentration amounts. Moreover, patients who had an ICB00 or DIU unilaterally inserted were included; therefore, the effect of binocular summation was not fully reflected. Although the degree of corneal astigmatism was not controlled, the corneal astigmatism was corrected with DIU, and the average value of SE after surgery was − 0.76 ± 0.37 D, and the median value was − 0.75 D, indicating that the astigmatism value was relatively unaffected. However, the effects of corneal astigmatism cannot be ignored. Finally, as pupil size data were unavailable, confirming the correlation between pupil size and QoV was not possible. In a follow-up study, a longer-term visual acuity evaluation targeting a larger number of patients with ICB00 implants in both eyes would increase the reliability of the study results. Furthermore, checking the photic phenomenon of ICB00 according to pupil size before and after surgery remains crucial.

Nevertheless, to the best of our knowledge, this is the first clinical study showing a correlation between the decenter amount of ICB00 and visual acuity. Additionally, this study is significant in that it is the first to multilaterally evaluate the performance of the ICB00 according to various preoperative ocular parameters such as angle alpha or SA. This result can be useful when the surgeon predicts the postoperative outcome based on the patient’s ocular parameters before inserting the ICB00 or DIU.

## Conclusions

In this study, we evaluated the performance of ICB00 preoperatively from various angles according to various ocular parameters. The larger preoperative SA corresponds to better visual acuity of ICB00 UNVA and UIVA. ICB00 generally has good UIVA and simultaneously maintains the QoV; however, patients with a large preoperative angle alpha (> 0.35 mm) may not be as satisfied as expected with postoperative UIVA. Furthermore, patients with a large ACD (> 3.4 mm) and younger patients may also have a photic phenomenon in ICB00; therefore, careful selection is recommended.

## Data Availability

The datasets used and/or analyzed in the current study are available from the corresponding author upon reasonable request.

## Abbreviations

IOLs: Enhanced monofocal intraocular lenses
SE: Spherical equivalent
CDVA: Corrected distant visual acuity
LogMAR: Logarithm of the minimum angle of resolution
AL: Axial length
ACD: Anterior chamber depth
WTW: White-to-white
RMS: Root mean square
ELP: Estimated lens position
UNVA: Uncorrected near visual acuity
UIVA: Uncorrected intermediate visual acuity
UDVA: Uncorrected distant visual acuity
